# The Mechanism of Hearing Loss in Paget's Disease of Bone

**DOI:** 10.1097/00005537-200404000-00002

**Published:** 2009-01-03

**Authors:** Edwin M Monsell

**Affiliations:** Department of Otolaryngology-Head and Neck Surgery, Wayne State University School of MedicineDetroit, MI, U.S.A.

**Keywords:** Sensorineural hearing loss, conductive hearing loss, presbycusis, Paget's disease of bone, quantitative CT, bone mineral density, auditory evoked potentials

## Abstract

**Objectives/Hypothesis:**

The mechanism of hearing loss (HL) in Paget's disease of bone was investigated. The present study was a systematic, prospective, controlled set of clinical investigations to test the hypothesis that there is a general underlying mechanism of HL in Paget's disease of bone and to gain additional insights into the auditory and otologic dynamics of this disease. Specific questions were 1) whether the mechanism is cochlear or retrocochlear and 2) whether the bone mineral density of the cochlear capsule is related to hearing levels.

**Study Design:**

Several double-blinded, cross-sectional, prospective, correlational studies were conducted in a population of elderly human subjects with skull involvement with Paget's disease versus a control population of elderly subjects free of Paget's disease. Demographic and clinical data were recorded. Longitudinal observations were made in subjects under treatment.

**Methods:**

Subjects were recruited from a Paget's disease clinic. Pure-tone auditory thresholds, word recognition, and auditory brainstem responses (ABRs) were recorded. The dimensions of the internal auditory canals were measured using computed tomographic (CT) images and digital image analysis. The precision, accuracy, and temporal stability of methods to measure the bone mineral density of the cochlear capsule and an adjacent area of nonotic capsule bone were validated and applied. Correlations were sought between hearing levels and cochlear capsule bone mineral density.

**Results:**

ABRs were recorded in 64 ears with radiographically confirmed Paget's disease involving the skull. Responses were absent in eight ears, all of which had elevated high pure-tone thresholds. ABRs were interpreted as normal in 56 ears; none were abnormal. The mid-length diameter and minimum diameter of the internal auditory canal of 68 temporal bones from subjects with Paget's disease were found to have no statistically significant relationship to hearing thresholds. The Pearson product-moment correlation coefficients (age- and sex-adjusted) in the group with Paget's disease involving the temporal bone were −0.63 for left ears and −0.73 for right ears for high-frequency air conduction pure-tone thresholds (mean of 1, 2, and 4 kHz) versus cochlear capsule density. Correlation coefficients (age- and sex-adjusted) between cochlear capsule density and air-bone gap (mean at 0.5 and 1 kHz) for the affected group were −0.67 for left ears and −0.63 for right ears. All correlations between hearing thresholds and cochlear capsule density in pagetic subjects were significant at *P* < .001. The regressions were consistent throughout the ranges of hearing level. There were no significant correlations between cochlear capsule mean density and hearing level in the volunteer subjects.

**Conclusions:**

The evidence supports the existence of a general, underlying, cochlear mechanism of pagetic HL that is closely related to loss of bone mineral density in the cochlear capsule. This mechanism accounts well for both the high-frequency sensorineural HL and the air-bone gap. Early identification, radiographic diagnosis of temporal bone involvement, and vigorous treatment with third-generation bisphosponates are important to limit the development and progression of pagetic HL.

## INTRODUCTION

Paget's disease is a disorder of osteoclasts. Its etiology is unknown. There is evidence of the involvement of a paramyxovirus 1 and genetic factors. 2 Pagetic osteoclasts are larger, and they are more active resorbers of bone by an order of magnitude or more. Progressively enlarging lytic lesions are formed. Osteoblasts react to the accelerated osteolysis of pagetic osteoclasts by forming new bone at a greatly accelerated rate. Often, this bone is poorly lamellated. When the rate of bone resorption is controlled by medication, the rate of bone formation is gradually reduced. Lytic areas are at least partially repaired, and the new bone formed has a normal lamellar appearance. 3 A bibliography on Paget's disease for health professionals is maintained by the National Institutes of Health Osteoporosis and Related Bone Diseases National Resource Center at http://www.osteo.org/research.asp.

Hearing loss (HL) is a prominent feature of Paget's disease of bone when the skull is involved. 4–7 In auditory studies, the majority of patients were found to have a high-frequency sensorineural HL and a low frequency air-bone gap. 5,6,8 The mechanism of HL is not well understood.

Because of the lack of an animal model for Paget's disease of bone, studies of the mechanisms of HL are limited to postmortem studies and in vivo clinical investigations. Before 1990, efforts to understand the mechanism of HL in many otologic disorders, including Paget's disease of bone, were limited to descriptive histopathologic studies, usually of a small numbers of cases as they became available for study. The histopathologic findings in human temporal bones with Paget's disease have now been described in more than 50 cases, including some with audiometric data. 7,9,10 Several mechanisms for the sensorineural HL were suggested in these studies, including compression of the auditory nerve 11 or vascular shunts. 7 It was suggested that the air-bone gap may be caused by stiffness changes in the soft tissue elements of the middle ear. 7 Epitympanic spurs and proliferation of fibrous tissue adjacent to the ossicles have occasionally been demonstrated. 5,12

In a differing analysis, Khetarpal and Schuknecht 9 were unable to demonstrate histopathologic correlates of HL in a thorough study of 26 temporal bones. Most remarkable was the finding that even in cases of severe HL, there was no correlation between the hearing threshold and the number or location of surviving hair cells. 9 The authors also showed no evidence of ossicular fibrosis or ankylosis to account for the air-bone gap. Khetarpal and Schuknecht proposed that “both the conductive and sensorineural components of the HL in Paget's disease are caused by changes in bone density, mass, and form that dampen the finely tuned motion mechanics of the middle and inner ears.”^9^ They were unable to offer direct evidence to support this suggestion.

The present study was a systematic, prospective, controlled set of investigations to test the hypothesis that there is a general underlying mechanism of HL in Paget's disease of bone and to gain additional insights into the auditory and otologic dynamics of this disease. A group of elderly subjects with Paget's disease involving the skull and a control group of elderly subjects without Paget's disease were recruited for several investigations.

Auditory brainstem responses (ABRs) were recorded to search for evidence of retrocochlear disease. The presence of normal ABRs would be taken as evidence of normal retrocochlear function and a cochlear site of lesion. Dimensional measurements were made of the internal auditory canal using digital image analysis from computed tomographic (CT) images. Correlations were sought between internal auditory canal dimensions and hearing levels (both high-frequency air-conduction thresholds and the low-frequency air-bone gap) to determine whether narrowing of the internal auditory canal or compression of the auditory nerve would account for hearing levels in subjects with Paget's disease of the skull.

Precise, accurate, stable techniques of quantitative CT (QCT) have been used to measure the mineral density of human bone in vivo. For this study, a QCT method was developed and validated to measure the bone mineral density of small volumes of bone in the skull, specifically the cochlear capsule. Subjects with Paget's disease involving the temporal bone were scanned. Correlations were sought between density values versus hearing levels. If statistically significant correlations were found, the hypothesis would be supported.

## METHODS

### Experimental Subjects

All subjects in this study gave informed consent according to procedures of the institutional review board (Human Rights Committee) and the principles of the Declaration of Helsinki. Forty-two subjects with Paget's disease of the skull were evaluated during the term of the study. The diagnosis of Paget's disease was established using standard clinical criteria, including medical history, physical examination, laboratory tests, and radiologic studies by an endocrinologist subspecializing in bone and mineral diseases. The diagnosis was confirmed by clinical evaluation of CT scans by a neuroradiologist. The duration of disease, severity of disease, and degree of cochlear involvement varied widely.

A priori exclusion criteria included systemic bone disease, prior middle ear surgery, and known extraneous causes of HL (otosclerosis, chronic otitis, classic Menière's disease, and mastoiditis). Subjects with diabetes, mild hypothyroidism corrected by hormone replacement, or with histories of noise exposure were not excluded.

Thirty-seven subjects with Paget's disease involving the skull were recruited for studies of ABRs and dimensional studies of the internal auditory canal. Subjects ranged in age from 40 to 87 years. The mean age was 68 years. There were 23 female and 14 male subjects. Nine (24%) of these subjects were black and 28 (76%) were white.

Thirty-three subjects (66 ears) were available for studies of ABRs. Of these, two left ears were excluded from analysis because of prior middle ear surgery, leaving 31 left ears and 33 right ears, or 64 total ears with ABR data for analysis.

Thirty-seven subjects were available for CT scanning for dimensional measurements. The processing of CT images included a data reduction step that resulted in inadvertent omission of the medial portion of the internal auditory canal from the image data set of one right and two left ears (total 3 ears). Dimensional measurements could not be made from these incomplete data sets, leaving 36 right and 35 left ears (total 71 ears) for analysis of dimensions in pagetic versus normal subjects.

Thirty-five patients with Paget's disease involving the skull were available for both studies of the bone mineral density of the cochlear capsule (QCT) and hearing levels. There were 21 female and 14 male subjects with Paget's disease involving the skull. Eight (23%) of these subjects were black and 27 (77%) were white. The 35 patients ranged in age from 40 to 87 years. The mean age was 68 years. Data from three ears from three subjects were excluded from the analysis, two ears for a history of ear surgery and another because of chronic mastoiditis. Data from the remaining 67 ears (33 left and 34 right ears) were analyzed.

### Volunteer Subjects

A control group of 22 volunteer subjects over age 50 was recruited from among the spouses of pagetic subjects and others. Two volunteers were excluded, one each for Ménière's disease and otosclerosis, leaving a total group of 20 subjects. There were 10 men and 10 women in the control group. One woman was of black descent. The remaining 19 control subjects were white. The mean age was 59 years.

Volunteers had no clinical history of Paget's disease and underwent determination of serum alkaline phosphatase to exclude Paget's disease. A neuroradiologist unaware of the clinical diagnosis reviewed each set of QCT images to confirm the presence of Paget's disease in each pagetic subject and its absence in the volunteers. Other exclusion criteria for volunteers were the same as for the pagetic subjects.

### Audiometry and Auditory Brainstem Responses

Pure-tone behavioral audiometric thresholds for air and bone conduction were determined at 2.5 dB increments. Because of reports that a high-frequency sensorineural HL and a low-frequency air-bone gap are characteristic in Paget's disease, 5,8,13,14 audiometric frequencies that reflected these two effects were selected. A high pure-tone average was calculated as the arithmetic mean of pure tone thresholds at 1, 2, and 4 kHz. The air-bone gap was calculated as the mean of air-conduction thresholds at 0.5 and 1 kHz minus the bone-conduction thresholds at those frequencies.

The ABR was recorded with a commercially available clinical evoked potentials system (Quantum 84, Cadwell, Kennewick, WA). Electrodes were applied with a conductant paste to the vertex (noninverting input Cz), left and right mastoids (inverting inputs M1 and M2, respectively), and at the midfrontal location (ground Fpz). Simultaneous ipsilateral and contralateral recordings were conducted to optimize the identification of component wave V. The bioelectric activity was differentially amplified (10^5^), filtered (100–3000 Hz), and averaged (1,500–4,000 samples).

Stimuli consisted of 0.1 ms unipolar electrical rectangular pulses. Either condensation or rarefaction clicks were used, depending on which yielded the higher quality waveform. The sound stimulus was produced by a piezoelectric transducer that was coupled to the ear by a 26 cm length of silastic tubing terminating in a foam earplug. The stimulus intensity was 75 dB nHL for those subjects with normal hearing sensitivity and 85 dB nHL for those subjects with hearing losses. The stimulus repetition rate was 21.3 pulses per second. Each recording was duplicated at least once and was superimposed so that an assessment of waveform stability could be made. The latencies of ABR components waves I, II, III, IV, and V were tabulated (when identifiable) as were the amplitudes of waves I, III, and V. The wave V/wave I amplitude ratio was calculated.

There is no universally accepted method of interpretation of ABRs. In this study, ABRs were interpreted as though they were diagnostic tests for retrocochlear disease (disease of the auditory nerve and central auditory pathways). It was reasoned that abnormal ABRs could be taken as evidence of abnormal auditory nerve function (as opposed to abnormal cochlear function) as the site of the auditory lesion. ABR findings were compared with normative data established for interpretation for retrocochlear disease. These data consisted of latency and amplitude values from 32 clinic subjects with normal hearing. ABRs were considered normal if values were within 2.5 SD of the normal hearing group. 15

If latency or amplitude values were not within 2.5 SD of the values of the normal hearing group, another level of analysis was applied. No specific “correction factor” in milliseconds of latency per dB of high frequency HL was used; however, the degree of HL in the higher frequencies and the steepness of the audiometric slope in these frequencies was assessed. In the presence of a high-frequency cochlear HL, the portion of the cochlea that gives rise to the transduction events that will result in the ABR is understood to be the domain that normally codes for middle- and low-frequency sounds. The traveling wave takes a finite amount of time (approximately 1.2–1.5 ms) to reach the middle- to low-frequency portion of the cochlear duct, producing a latency shift, which is reflected in the ABR in subjects with high-frequency sensory HL. Thus, measured latencies that exceeded the reference values by the time expected based on the degree of high-frequency HL were interpreted as normal (for the degree and configuration of the HL) and consistent with intact auditory nerve function. 15

### Dimensional Measurements

**Fig. 1:**
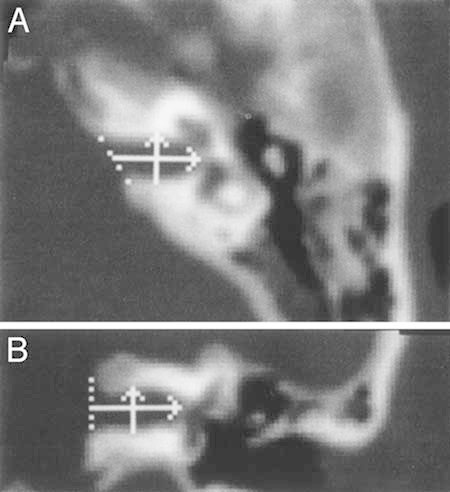
(**A** ) Axial and (**B** ) coronal computed tomography images of typical pagetic temporal bone showing placement of lines for dimensional measurement. 15 (Monsell EM, Bone HG, Cody DD, Jacobson GP, Newman CW, Patel SC, Divine GW. Hearing loss in Paget's disease of bone: evidence of auditory nerve integrity. *Am J Otol* 1995;16:27–33.)

Measurement of the dimensions of the internal auditory canal was accomplished with digital image processing. In step 1, a perpendicular pair of planar CT images (axial and coronal) was selected from the three-dimensional data set using digital image analysis software developed for this purpose. 16 The axial plane was selected first and included a section of maximal diameter throughout the internal auditory canal. The vestibule and portions of the cochlear and vestibular labyrinths were also included in this sectional image. The line of rotation between the axial and coronal planes was the line passing through the center of the internal auditory canal (Fig. 1).

In step 2, the length, the mid-length diameter, and the minimum diameter of the internal auditory canal in the axial and coronal planes of each temporal bone were measured. To simplify data analysis, the two sets of data (axial and coronal) for length and mid-length diameter were averaged. The smaller of the two minimum diameters (axial and coronal) was taken to identify all cases where auditory nerve compression might occur. Left and right ears were analyzed separately.

The precision of the dimensional measurements was tested in two ways. Step 2 was repeated for each pair of axial and coronal images in eight temporal bones at least 24 hours after the initial set of measurements (24 pairs of observations). Steps 1 and 2 were also repeated in eight temporal bones each (24 pairs of observations). During each second set of measurements, the operator was unaware of (i.e., “blinded” to) the first result. For the length and mid-length diameter, the average coefficients of variation were less than 3% for the first test of precision described above and less than 5% for the second test. The measurement of the minimum diameter was somewhat less precise, with a coefficient of variation of 5% for the first test and 11% for the second. 15

### Radiology

Much of the present study depended on the development and validation of the radiologic method of measuring bone mineral density of the cochlear capsule using a particular type of QCT. 17

The method of measuring bone mineral density of the cochlear capsule used in this study was a modification of a technique originally used to measure the regional bone mineral density in human vertebral bodies in vivo. 17 A specific convolution filter (mathematical algorithm to calculate the image from raw data) was applied to optimize the accuracy and precision of density measurements without sacrificing the high quality of the images. The technique resulted in a three-dimensional array of values expressed as physical density (e.g., mg/cm^3^). The amount of radiation exposure was similar to that of conventional CT.

**Fig. 2:**
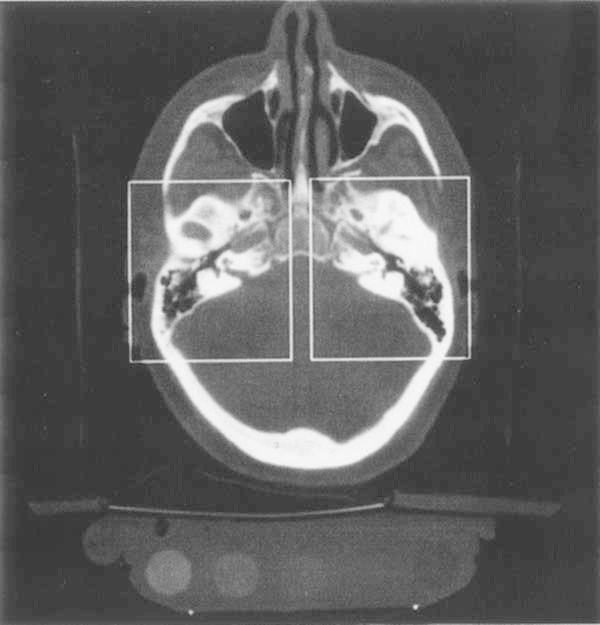
Axial quantitative computed tomography scan image of the head showing the calibration device beneath the head. White squares outline the subregions sampled (128 × 128 pixels). 16 Reprinted from *Hearing Research*, Vol 83, Monsell E, Cody D, Bone H, et al., Hearing loss in Paget's disease of bone: the relationship between pure-tone thresholds and mineral density of the cochlear capsule, Pages 114–120, Copyright 1995, with permission from Elsevier.

The scan protocol used an x-ray tube voltage of 130 kVp, 100 mA current, 4 seconds exposure time, and a 1 mm^2^ focal spot. The head holder incorporated a solid bone-mineral calibration phantom marketed by Image Analysis (Irvine, CA). This device had a graded series of five density standards consisting of hydroxyapatite embedded in a plastic resin (Fig. 2). Thirty to 50 scans with a slice thickness of 1 mm were taken through the temporal region of the skull of each subject in the axial plane. A scan diameter of 30 cm was reconstructed to 512 × 512 voxels, producing voxels of dimensions 0.585 × 0.585 × 1.0 mm. A subregion of 128 × 128 voxels was selected for analysis (Fig. 2). Measurement resolution was determined to be 1.6 mm in each dimension, providing a sample volume of 0.004 cm^3^. This method has previously produced measurements with noise of 0.7 to 1.3%, accuracy of 2.7%, and serial precision in vitro of 0.2%. 15 The long-term precision was monitored by use of a solid calibration standard (Image Analysis, Irvine, CA). A very stable result for a period of almost 4 years was accomplished. 16

To assess measurement accuracy, four cochlea-shaped plastic density standards were inserted into the temporal bones of a skull phantom. Measurements were confirmed to be linear through the range of densities encountered in Paget's disease. Measurements of the mineral density of normal cortical bone (1,850 mg/cm^3^) were consistent with the results of other laboratories. 18

### Image Analysis

**Fig. 3:**
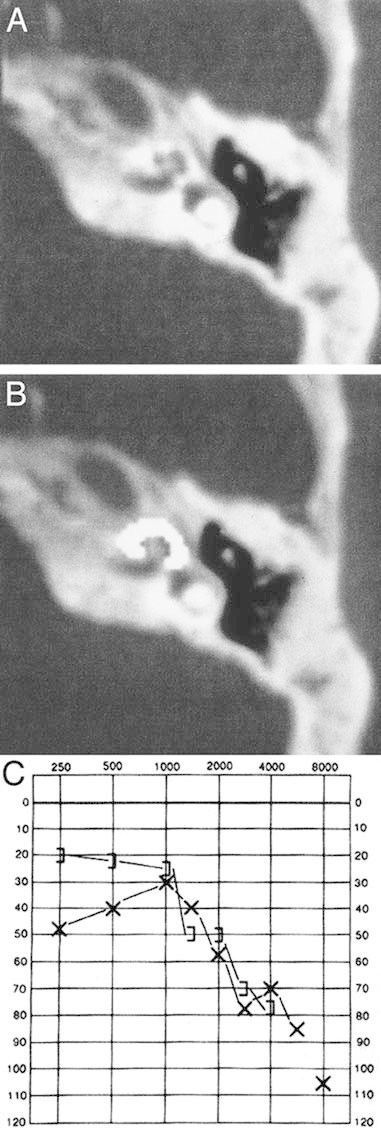
(**A** ) Example of a planar quantitative computed tomography image from a 66-year-old subject with Paget's disease moderately involving the left temporal bone. (**B** ) The same image showing the cochlear capsule regions of interest defined for this temporal bone. (**C** ) Air and bone conduction audiogram from the same subject and ear. 19 (Monsell EM, Cody DD, Bone HG. Measurement of regional bone mineral density: a new techniqe for the evaluation of the temporal bone. *Am J Otol* 1993;14:455–459.)

A custom software package with a trilinear interpolation algorithm was used to translate and rotate the image data so that a thin section of the temporal bone oriented in a standard plane could be selected for analysis interactively on a computer workstation. This plane incorporated the lateral semicircular canal and a near mid-modiolar section of the cochlea (Fig. 3A). 19

Regions of interest were constructed from these sectional images to sample the bone density in the cochlear capsule (Fig. 3B). 19 Regions of interest were defined with particular care to correspond to the structure of the cochlear capsule and to avoid overlapping the area of study with the air-containing space of the middle ear. Such overlapping could result in errors caused by partial volume effects. Three-dimensional regions of interest that included the entire cochlear capsule were used for studies of treatment response.

Two studies were conducted to assess the consistency of image analysis. First, a second region of interest was constructed on the original image section at least 24 hours after constructing the first region of interest, and the density measurements from each region of interest were compared. In this manner, the error attributable to selection of the regions of interest was demonstrated to be 0.7%. 16 In the second study, the image section that passed through the lateral semicircular canal was reselected, and a region of interest on this second sectional image was defined. The error attributable to the complete process of image analysis was found to be 1.5%, including selection of the lateral canal plane and construction of the regions of interest. 16

### Statistical Analysis

The relationship between dimensions and hearing measures was assessed by correlations. Internal auditory canal dimensions were compared between the Pagetic subjects and controls by use of t tests and analysis of covariance. In each case, adjustment was made for the covariates age and sex.

Regression methods were used to examine the relationship between hearing thresholds and mean bone mineral density of the cochlear capsule. Linear regression relationships and Pearson product-moment correlations were computed to assess the simple associations. Analysis of covariance was used to compute partial correlation coefficients, adjusted for the covariates of age and sex. A Bonferroni adjustment to the *P* values considered statistically significant ([0.05]/2 = 0.025 or less) was performed to account for the two measures of hearing that were tested.

## RESULTS

### Auditory Brainstem Responses

Eight ears in five subjects had no recordable auditory evoked responses caused by low hearing levels. ABRs from the remaining ears were normal, indicating that there was no electrophysiologic evidence of impairment of auditory nerve transmission.

### Dimensional Measurements

Results are summarized in Tables I and II. 15 Correlations were sought between the length, mid-length diameter, and minimum diameter versus high pure-tone threshold average (1, 2, and 4 kHz) and versus the low-frequency air-bone gap (0.5–1 kHz). No association was found between internal auditory canal dimensions and hearing level in the volunteer subjects. There were significant correlations of length versus both high-tone average and air-bone gap (Table II). t tests did not demonstrate that any of the dimensions were significantly different between pagetic and control groups.

**TABLE I:** Mean Internal Auditory Canal Dimensions

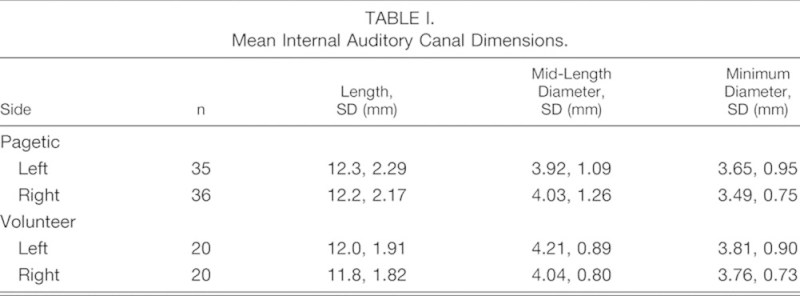

**TABLE II:** Internal Auditory Canal Dimensional Correlations in Pagetic Subjects

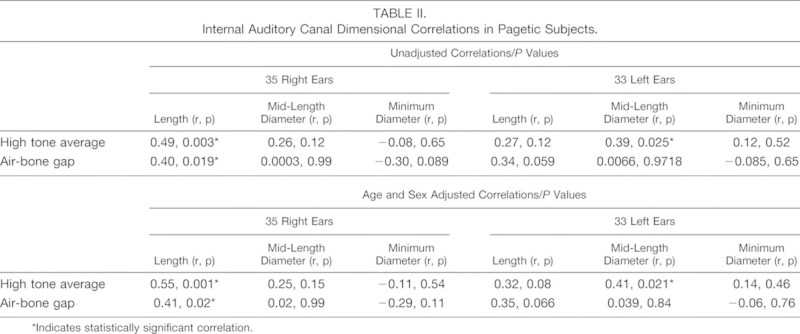

There were no significant correlations between hearing level and the minimum diameter in either the pagetic or the volunteer subjects. There were significant correlations between mid-length diameter and high-tone average, but these were weak and were significant only in left ears (Table II). In one extensively pagetic temporal bone, the minimum coronal diameter was 1.17 mm (pure-tone average at 0.5, 1, 2, and 4 kHz = 42 dB), the smallest diameter measured in this study. The corresponding diameter on the opposite side was 1.76 mm (pure-tone average = 42 dB).

### Comparison of Left versus Right Ear Cochlear Capsule Density

**Fig. 4:**
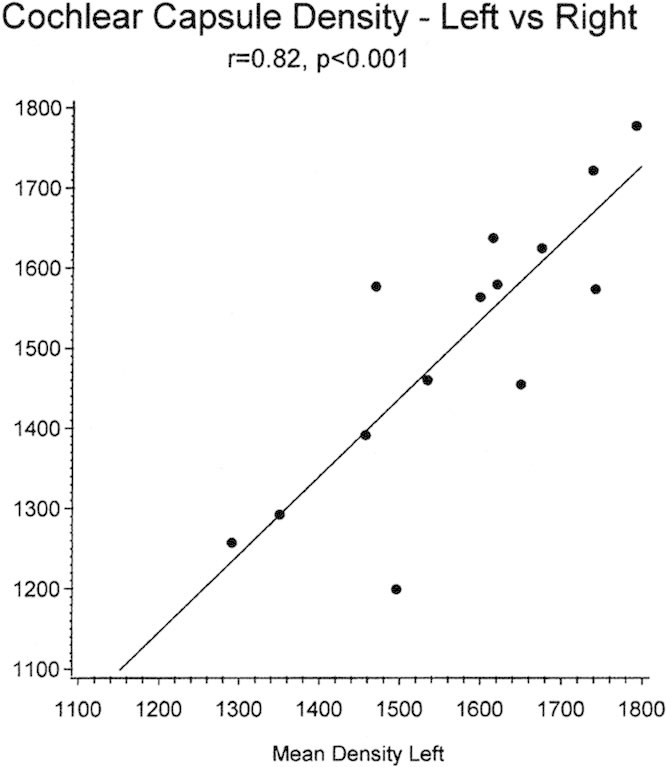
Scatterplot of cochlear capsule density in subjects with Paget's disease involving the temporal bone, left versus right ears.

Left and right cochlear capsule densities were highly correlated (r = 0.82, *P* < .001). The regressions were consistent throughout the ranges of cochlear capsule density (Fig. 4). These findings confirm clinical observations that pagetic involvement of the skull is most commonly symmetrical.

### Relationship of Bone Mineral Density of the Cochlear Capsule to Hearing Levels

**Fig. 5:**
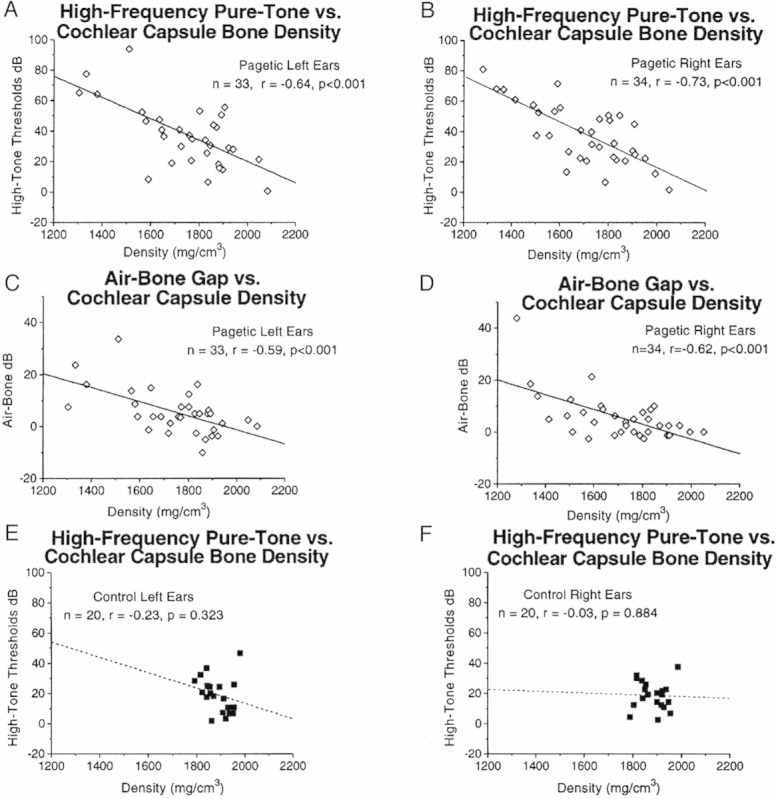
(**A, B** ) Scatterplots of the correlations of high-frequency pure-tone hearing thresholds by air conduction versus mean cochlear capsule bone mineral density in pagetic ears. Note the strong correlation for all ranges of hearing and bone density. (**C, D** ) Scatterplots of the correlations of air-bone gap versus mean cochlear capsule bone mineral density. (**E, F** ) Scatterplots of high-frequency pure-tone hearing thresholds by air conduction versus mean cochlear capsule bone mineral density in volunteer subjects show no significant relationship. 16 Reprinted from *Hearing Research*, Vol 83, Monsell E, Cody D, Bone H, et al., Hearing loss in Paget's disease of bone: the relationship between pure-tone thresholds and mineral density of the cochlear capsule, Pages 114–120, Copyright 1995, with permission from Elsevier.

Significant Pearson product-moment correlations were found between high-frequency pure-tone air conduction hearing thresholds and mean bone mineral density of the cochlear capsule in 33 left ears and 34 right ears of the 35 Paget's disease subjects both with and without correction for age and sex (Table III) 16 (data representing three ears were not included because of exclusion criteria). Scatter plots (Fig. 5) illustrate the consistency of the regression functions throughout the range of the high-frequency pure-tone hearing levels. Multiple regressions showed that correlations between cochlear capsule density and hearing threshold at individual frequencies from 1 through 4 kHz were not substantially different from each other.

**TABLE III:** Correlations Between Hearing Thresholds and Mean Density of the Cochlear Capsule

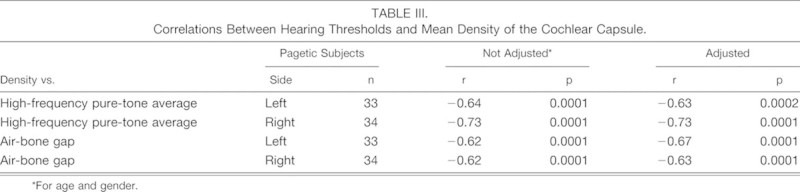

Significant correlations also were found between mean cochlear capsule density and the air-bone gap in the pagetic subjects (Fig. 5, C and D). In contrast, there were no significant correlations between cochlear capsule mean density and the high frequency pure-tone hearing levels in the volunteer subjects (Fig. 5, E and F). Because normal subjects are not expected to have conductive HL or air-bone gaps, it is questionable whether calculation of correlations involving the air-bone gap in the volunteer subjects would be meaningful. As expected, there were no significant correlations between cochlear capsule mean density and the air-bone gap in the volunteer subjects.

The robustness of the correlations was explored by examining the effect of risk factors for HL. The effect of risk factors, including noise exposure (by history) and diabetes mellitus, was examined by comparing selected pagetic subjects without risk factors (n = 22) to those with risk factors (n = 13). There were no significant differences between the two groups in correlations of either the high pure-tone threshold level or the air-bone gap. There was no apparent relationship between sex and high-frequency pure-tone thresholds; however, men may have had a smaller air-bone gap by 3.8 dB for left ears or 3.7 dB for right ears (*P* = .10 and *P* = .03, respectively) after adjustment for age and mean bone density of the cochlear capsule. 16

## DISCUSSION

The ABR results are consistent with intact auditory nerve function in this group of subjects with Paget's disease of the skull. Correlational data in right temporal bones suggested that the length of the internal auditory canal might be related to high-tone hearing level. However, this association was less clear on the left side and appeared qualitatively weaker for conductive losses compared with sensorineural losses (Table II). The biologic or clinical significance of these possible weak associations is unclear, although it is conceivable that extensive Paget's disease is correlated with bossing of the porus acusticus in some cases of moderate to advanced disease and has been observed histologically. 9

Olivares and Schuknecht 20 measured the diameters of the internal auditory canal in 435 histologically prepared temporal bones. Specimens with evidence of bone disease, including Paget's disease, were specifically excluded. One hundred forty-four ears with sensorineural HL were purposely included. The authors found no relationship between the width of the internal auditory canal and hearing level. Ten subjects had canal widths of 3 mm or less and average pure tone thresholds of 30 dB or better in frequencies 0.5, 1, and 2 kHz. The authors concluded that it is doubtful that narrowing of the internal auditory canal is associated with HL in nonpagetic sensorineural HL. The differences between the measurements of the mid-length diameter of the internal auditory canal from digital radiographic data (mean of all mid-length diameters in pagetic subjects = 3.97 mm) and the 6% smaller measurements derived from histologic preparations (mean of 435 subjects = 3.72 mm) by Olivares and Schuknecht 20 may be caused by shrinkage of tissue during histologic processing.

The possibility of partial auditory nerve compromise in occasional instances cannot be excluded absolutely; however, our radiologic and electrophysiologic data indicate that auditory nerve function is intact in most cases of HL associated with Paget's disease of bone. The results support the hypothesis that the primary effect of Paget's disease on hearing is on the cochlea.

The results with QCT show the feasibility of determining regional bone mineral density in the temporal bone, including small structures such as the cochlear capsule. Although the linear resolution of measurement is 1.6 mm, voxel dimensions of 0.585 mm enable accurate measurement of mean density of small regions by sampling multiple voxels, typically 60 to 100 for each cochlear capsule. Errors caused by voxels that include in their volume different structures of adjacent regions (partial volume effect) were minimized but not completely eliminated.

A strong and statistically significant relationship was demonstrated between the bone mineral density of the cochlear capsule and both the high-frequency pure-tone air-conduction thresholds and the air-bone gap in subjects with Paget's disease of the skull. There does not appear to be an inflection in the regression line or suggestion of a threshold phenomenon in this relationship. This consistency of the regression throughout its range suggests that the effect of Paget's disease on hearing is a continuous, graded process.

The bone of the normal cochlear capsule is lamellar bone with few haversian canals and vascular elements and thus consists of dense, compact bone tissue. 21 Consequently, bone lysis by Paget's disease would result in density values less than normal, whereas bone sclerosis could only thicken the cochlear capsule, not increase its mineral density. In accordance with this principle, in this study, pagetic involvement of the skull was associated only with reductions in cochlear capsule density or normal values, depending on the degree of the pagetic effect.

The findings in the studies reported here strongly support the hypothesis that there is a general underlying mechanism of HL in Paget's disease of bone. The finding of normal ABRs supports the cochlea as the site of the pagetic lesion rather than the auditory nerve. The lack of evidence for nerve compression further supports the cochlear hypothesis and demonstrates that there is very little distortion of consequence in the shape of the temporal bone, except perhaps in rare, extreme cases. The demonstrated relationship between cochlear capsule mean density and hearing levels also supports the cochlear hypothesis and the hypothesis of a general underlying mechanism. This relationship also suggests that alteration of bone mineral density may be close to the mechanism of HL.

These correlational findings are not necessarily in direct conflict with the findings of earlier histologic studies of pagetic temporal bones. The possibility of additional mechanisms, particularly in advanced cases, is not excluded.

Audiometric data, particularly data involving the determination of bone-conduction thresholds, are subject to considerable variability, especially in elderly subjects. The subjects in this study were older than the general population. Consequently, it must be acknowledged that the strength of the correlations is remarkable. The positive finding of correlations between cochlear capsule mineral density versus hearing levels supports the validity of the negative finding of no significant correlations between dimensional measurements of the internal auditory canal and hearing levels.

The idea that an air-bone gap can exist without a conductive HL in the manner usually understood may be difficult to accept. Khetarpal and Schuknecht 9 originated the concept with their report of no fibrosis or ankylosis involving the ossicles, their articulations, or suspensory ligaments. Currently, Paget's disease is the only instance known of an air-bone gap that does not indicate an impairment of the sound conducting mechanism. The implication is that the air-bone gap is a phenomenon of the acoustical mechanics of hearing by bone conduction. We are led to suspect that the air-bone gap may be caused in some way by enhanced conduction of low-frequency sound energy by pagetic bone. The strong correlations between bone mineral density of the cochlear capsule and the air-bone gap suggest that the enhancement occurs primarily in the cochlear capsule, although enhancement may also occur in pagetic bone of the skull base more generally.

Pagetic bone has been observed to change shape. Gradual bowing of the weight-bearing long bones is common when they are involved. HL in Paget's disease does not seem to be related to changes in the shape of the skull or inner ear. Platybasia, a flattening and widening of the skull base, occurs only in the most advanced cases of pagetic involvement of the skull, whereas HL occurs with almost any degree of involvement of the temporal bone. The dimensional analysis of pagetic temporal bones in this study did not show changes in the internal auditory canal. Also, we did not observe qualitative changes in the shape of the cochlea.

Treatment with the newer bisphonsponates provides rapid arrest of the pagetic process and long-term control. 22 Acute spinal neuropathy caused by involvement of the bone adjacent to the spinal cord recovers in hours to days during bisphosphonate treatment, possibly because of a vascular steal phenomenon or the effects of toxic cytokines on neural structures. 23 Nevertheless, it has been the common observation, confirmed in this study and the author's clinical experience, that pagetic HL does not recover despite vigorous treatment. Consequently, spinal neuropathy is not a suitable model for pagetic HL. The possibility that pagetic bone releases toxic cytokines that impair cochlear function irreversibly but without resulting in loss of hair cells was not addressed by the study and cannot be excluded. Also, spiral ligament atrophy, which occurs prominently in otosclerosis, is not a typical feature of pagetic HL.

Histologic studies show that the pagetic process involves the entire cochlear capsule in essentially all cases observed. This finding suggests that the entire capsule is involved early. A reasonable inference from the cross-sectional data of the study is that bone mineral density is gradually lost as the disease progresses. It may be suspected that continuous remodeling of pagetic otic capsule bone results in progressive sensorineural HL, predominantly in the higher frequencies. One is tempted to speculate that perhaps Paget's disease reveals the normal auditory function of otic capsule bone. This bone normally does not undergo remodeling. When it does undergo remodeling in Paget's disease, a gradual decline occurs in auditory function.

Sensory transduction in the cochlea is a process of interaction between mechanical (acoustic) and electrical events. It appears that conformational change in membrane-bound ion channels may be induced by mechanical deformation. The flow of ions across hair cells is dependent on the maintenance of the “silent” current. 24 This current flow may be reduced in pagetic bone. It is also possible that acoustic energy is absorbed by pagetic bone, resulting in lower amplitude displacements of the basilar membrane in response to sound.

This study demonstrates the importance of studying a disease process in a systematic, prospective manner. It also demonstrates the difficulties of doing so. Progress requires the combination of a large group of subjects, advanced investigative techniques that are validated, and a multidisciplinary approach. Bone disorders that affect hearing, such as otosclerosis, are among the most common otologic disorders. Progress in understanding disorders of bone that affect hearing is hampered by a general lack of knowledge of the biology of calcified tissue, by the lack of more detailed knowledge about the unique features of otic capsule bone, and the lack of a suitable animal model. It is likely that each disease process (Paget's disease, otosclerosis, osteogenesis imperfecta, etc.) will prove to involve different mechanisms of HL. Despite the difficulties, the effort is worthwhile. Not only can more be learned about the causes, prevention, and treatment of HL, but we also may gain insight into normal auditory function.

## CONCLUSIONS AND CLINICAL RECOMMENDATIONS

The findings in this study support a new view of the mechanism of HL in Paget's disease of bone and a new approach to clinical management of skull involvement:

1. There is a general underlying mechanism of HL in Paget's disease.
2. The mechanism of HL is cochlear.
3. The loss of bone mineral density in the cochlear capsule is a marker of disease effect and is close to the mechanism of both the sensory HL and the air-bone gap.
4. The strong correlation between the bone mineral density of the cochlear capsule and air-bone gap supports the suggestion that the air-bone gap in Paget's disease is not caused by pathology of the ossicular chain but by alteration of the acoustical mechanics of the ear. This alteration may include facilitation of bone conduction of sound.
5. Treatment that normalizes biochemical markers of active disease does not result in clinically significant improvement in hearing levels.
6. The pagetic process and associated HL are established well before signs appear in the facial skeleton. Consequently, it is not appropriate to withhold treatment until such signs appear.
7. HL caused by Paget's disease of bone is gradually progressive but potentially preventable with early diagnosis and vigorous treatment. Third-generation bisphosponates are highly effective in controlling the pagetic process.
8. Pagetic HL should be suspected in any middle-aged or elderly person, especially if hearing levels progress more rapidly than would be suspected in presbycusis or if a low-frequency air-bone gap is present. 22,25 A serum alkaline phosphatase level can exclude the disease if normal or lead to definitive diagnostic evaluations if abnormally elevated.
